# Why and how the humanities matter for sustainability transformations: a humanities-sustainability science bridging perspective

**DOI:** 10.1057/s41599-026-09457-1

**Published:** 2026-11-05

**Authors:** Andra-Ioana Horcea-Milcu, Tamara Bodden, Martin Böhnert, Mateo Chacón Pino, Agnieszka Komorowska, Paul Reszke, Angela Schrott

**Affiliations:** 1 Kassel Institute for Sustainability, University of Kassel, Kassel, Germany; 2 Faculty of Humanities and Cultural Studies, University of Kassel, Kassel, Germany; 3 Department for German Studies, University of Koblenz, Koblenz, Germany; 4 Institute of Philosophy, University of Kassel, Kassel, Germany; 5 Documenta Institut, Kassel, Germany; 6 Department of Architecture, HafenCity University, Hamburg, Germany; 7 Department of Romance Studies, University of Kassel, Kassel, Germany; 8 Deutsches Institut, Faculty of Philosophy and Philology, Johannes Gutenberg University Mainz, Mainz, Germany

**Keywords:** Complex networks, Cultural and media studies, History, Language and linguistics, Literature, Philosophy, Religion

## Abstract

Hitherto dominant modes of researching and governing transformation(s) are insufficient for society, including science, to adequately respond to the current polycrisis. For science to help guide, steer, and enable transformative change, all disciplines, including the humanities, are needed. The contemporary research landscape on sustainability transformation is diverse yet fragmented. Until very recently, the humanities have mostly occupied a peripheral position within the mainstream transformative change research, in part because their relevance appears less pronounced within positivist and linear frameworks. We aim to reduce this fragmentation by highlighting how concepts and skills from the humanities are applied to research on sustainability transformations. This paper is intended for a diverse readership: sustainability scientists seeking to deepen their transformative impact and humanities scholars looking for interdisciplinary entry points into the discourse on sustainability transformations. We highlight why and how the humanities matter for sustainability transformation by focusing on their contributions to systems, target and transformation knowledge. In particular, we argue that the humanities are uniquely suited to contribute target and transformation knowledge. They support the “how” of transformative change and enable the co-creation of visions and solution options that match the complexity of sustainability challenges. Through nine interrelated points, we illustrate how the humanities are active in co-creating and are further positioned to enrich visions and pathways for sustainability transformation(s).

## Building a bridge from both sides: contributions of the humanities and sustainability science to support sustainability transformations

In recent decades, most visible sustainability discourses have operated under the assumption that scientific and technological solutions alone will help humanity out of polycrisis and move its trajectory toward sustainability. Environmental sciences and biodiversity conservation also played a visible role, with numerous studies initially focusing on maintaining the adaptability and resilience of ecosystems (Holling, 1973; Elmqvist et al. 2003; Folke et al. 2004). Quite rapidly, the social sciences gained importance, albeit the uptake of insights has been uneven, with a tendency to prioritize those fitting within a positivist frame emphasizing generalizable patterns and empirical observation (Hulme, 2011; Castree et al. 2014). The mainstream sustainability discourse moved towards understanding and managing human-environment dynamics, often conceptualized as social-ecological systems (SES) (Folke et al. 2005; Walker and Salt, 2006), human-environment systems (Turner et al. 2003) or coupled human and natural systems (CHANS) (Liu et al. 2007). On the whole, when faced with the imminence of the sustainability crises, the natural sciences, technology, economics, political sciences and sociology were invited to provide explanations and even potential solutions.

In parallel, at the end of the 90 s, sustainability science emerged as a new, distinct, problem-driven, solution-oriented, normative scientific program aspiring to operate at the science|practice interface (Kates et al. 2001; Clark and Dickson, 2003; Martens, 2006; Clark, 2007). It was mandated to integrate multiple interdisciplinary contributions to understand human-nature relationships (the prevailing descriptive-analytical stream) and to actively transform them (the transformative stream). As such, sustainability science occupies a rather central position in the transformation research landscape. Initially designed to be ‘different’ (Clark and Dickson, 2003), sustainability science has been expected to renegotiate the science-society relationship by mediating new, often inter- and trans-disciplinary, modes of knowledge (co-)creation (Gibbons, 1999; Lang et al. 2012; Clark and Harley, 2020; Lang and Wiek, 2022). Very recently, the focus on relationships and their constitutive role in entities has been termed the “relational turn” in sustainability science (West et al. 2020; Walsh et al. 2021; Haider and Rieser, 2025). Together with the rise of critical sustainability science (Rist et al. 2024; Longo et al. 2025), it marks promising entry points for the humanities in sustainability science and in sustainability research more broadly. The relational turn offers a sensible alternative and complementary approach to the predominant substantialist view in the history of sustainability science, underpinned by a dualist ontology (a fundamental separation between distinct domains, e.g., nature and culture) (Scarano, 2020; Rist et al. 2024). Increasingly advanced in the past years (Vasquez-Fernandez and Ahenakew pii tai poo taa, 2020; West et al. 2024; Gould et al. 2025; Hertz et al. 2025), process-relational perspectives challenge conventional separations between the ‘social’ and the ‘ecological’, and frame sustainability transformations as open processes giving prominence to humanities-rooted ontologies (Hertz et al. 2025).

Today, there is broad agreement that for science to guide and steer sustainability transformations, it needs to mobilize its full breadth, including the natural and social sciences and the humanities (IPBES, 2024a). Instead of seeking a single “correct” mode of conducting research on or for sustainability transformations, a plurality of approaches should be acknowledged as a collective strength of the research community at large, rather than as a source of fragmentation (Dillon and Wals, 2006). Despite attempts to bridge divides and attenuate hegemonies (Wilson, 1999; Kagan, 2009; Lang et al. 2017; Vadrot et al. 2018; Moon et al. 2019; Pérez-Hämmerle et al. 2025), fragmentation or the prevalence of certain disciplines persists (Fischer et al. 2007; Nelson, 2025). Even as the humanities increasingly engage with the crisis of the Anthropocene (Alonso, 2015; Holm et al. 2015; Horn and Bergthaller, 2020; Gabriel et al. 2022), their knowledge remains comparatively less visible within transformation-oriented research (Simeone, 2018). This is reflected, for example, in the distribution of sustainability institutes: many explicitly bring together natural sciences and/or engineering and/or social sciences, while comparatively few foreground the humanities or integrate them on equal footing, with the exception of sustainability-oriented institutes in the social sciences and humanities. A similar pattern can be observed in academic events on sustainability transformation: although humanities scholars are occasionally invited as panelists and some conferences include humanities-rooted sessions, these remain the minority. In much of the remaining program, competencies typically associated with the humanities, such as discourse competencies (studying how power relations are enacted through communication practices), narrative framing (structuring issues through stories and interpretive lenses), ethical reasoning (reflecting on values and responsibilities), or affective-cultural sense-making (interpreting culturally mediated meanings and emotions), may be referenced, yet without direct or explicit engagement with humanistic scholarship.

While ecology, social sciences, and technical sciences are often part of scientific responses to shaping more just and sustainable futures, humanities perspectives continue to occupy a more peripheral position in mainstream discourses on transformative change (ISC, 2021; Poliseli and Caniglia, 2024). Their insights into meaning, moral ambiguity, and the role of culture and imagination in transformation remain largely untapped. This is partly because their normative depth (the capacity to evaluate what is desirable or ethical) and their interpretive and reflexive approaches, which go beyond measuring observable variables to uncover underlying meanings and cultural codes, do not easily align with positivist scientific worldviews. In such frameworks, valid knowledge is often understood to proceed linearly from empirical observation to inform causal explanation and prediction (Palsson et al. 2013; Creswell, 2014; Moon and Blackman, 2014; Mäkinen-Rostedt et al. 2023; Turnhout, 2024). For example, the term “social-ecological transformations” may be misleading, as it primarily evokes the social and ecological sciences, potentially suggesting that these are the main pillars of thinking and acting towards transformative change. However, dominant and established modes of research and, ultimately, of governing transformations are not sufficient to realize fundamental, system-wide shifts in views, structures, and practices (IPBES, 2019). All options are needed, and each has its own assumptions about what drives and how transformative change happens (Bennett et al. 2024). Yet any single perspective has inevitable blind spots, as transformations are both external and internal, structural and relational, systemic and personal (Bennett et al. 2024).

## Aim and background

The goal of this paper is to make a case for why and how the humanities matter both for research on sustainability transformations (the more descriptive-analytical transformation research) and especially for research for sustainability transformations (the more interventional transformative research) (Linnér and Wibeck, 2019; Horcea-Milcu et al. 2024). Our intent is fourfold: i) to increase the accessibility of humanities perspectives for sustainability scientists working in the transformation field, particularly those more accustomed to natural or environmental social science approaches or trained within these disciplines; ii) to reduce fragmentation within the wide sustainability transformation research landscape by strengthening, in the case of this paper, the relationships between humanities contributions and sustainability science contributions for sustainability transformations; iii) to foreground and translate key humanities contributions through sustainability science heuristics, addressing the sustainability science (Clark, 2007) community in particular, as well as sustainability researchers more broadly; iv) to navigate the tension between the risk of “getting «bogged down» by philosophical debates in crisis situations” (West et al. 2020: 306), and “the trepidation at the thought of engaging with the interventionist” (West et al. 2020: 306) stream of sustainability science, because of indiscriminate aggregations, necessary deconstructions, uncritical stances, unacknowledged power dynamics, inexplicit situatedness, and open ethical questions. In so doing, we acknowledge that many knowledge-to-action models in sustainability science operate with a necessarily reductive logic that may act as a barrier to alternative approaches, yet we also recognize their practical utility and heuristic power in structuring and mobilizing insights for transformation. Hence, we start from the premise that scholars of sustainability transformation often draw on sustainability science. In line with the transformative branch of sustainability science (Wiek and Lang, 2016), our primary aim is not to analyze the underlying causes of the humanities’ underrepresentation within the sustainability transformation landscape and its documented structural constraints (Salmela et al. 2025). Rather, we seek to make visible, particularly from a sustainability science perspective, the ways in which the humanities can, or already do, contribute to sustainability transformation debates and knowledge production.

Most co-authors of this perspective paper have backgrounds in the humanities (history, literary studies, linguistics, art history, philosophy, theology, religious studies and pedagogy). We consider the humanities to be a diverse and established—unlike the comparatively emerging field of sustainability science—disciplinary field comprising the aforementioned disciplines alongside others such as cultural studies, gender studies, and communication sciences. While recognizing that humanists do not form a monolithic block, they share a primary interest in “an understanding of human reactions to events and the meanings humans impose on experience as a function of culture, historical era, and life history”, based on “written texts and human behaviors gathered under conditions of minimal control” (Kagan, 2009:4). The humanities hence offer resources for interpreting the human condition, its values and narrative formations (Bod, 2013). As our intent is to make such humanistic perspectives accessible to the sustainability (transformation) research community at large, rather than to humanists themselves (“preaching to the converted” in Lang et al. 2017:877), an aggregated representation of the humanities serves this purpose.

This perspective paper is led by a sustainability scientist with: i) expertise in transdisciplinary co-creation of knowledge as one of the knowledge production modes of sustainability science, ii) no formal training in the humanities, iii) multiple experiences working collaboratively with humanists over the past five years in academic and science-policy settings, iv) ongoing experimentation in an integrator role (Hoffmann et al. 2022). The first author acknowledges that approaching humanities scholarship from outside the field inevitably entails a bounded perspective. The paper was co-written iteratively, beginning with eight semi-structured interviews on how and why the humanities matter for sustainability transformations. Interviews were conducted in a conversational mode by the first author in 2023 and 2024 with humanities-trained academic peers. Insights from the interviews were first used to populate the three types of knowledge heuristics and were then inductively organized within knowledge types into nine overarching themes. Themes were subsequently corroborated with literature from both sustainability science and the humanities: sustainability science references were primarily drawn on by the first author, while humanities references were contributed by the co-authors, reflecting their respective disciplinary expertise. This corpus was further informed by insights from the knowledge-gap analysis on building capacities for transformative change performed within the IPBES Transformative Change Assessment (Box 9 in IPBES, 2024a). The paper was refined through successive drafts, including checking with interviewees on the themes derived from their input, and based on oral presentations, including feedback received at the Sustainability Science summit 2025, organized by the German Committee Future Earth (Deutsches Komitee für Nachhaltigkeitsforschung in Future Earth, DKN) in February 2025. To offer a bridging perspective, the referenced literature spans contributions from both the humanities and sustainability science, with a particular emphasis on recent publications that reflect the emergent and rapidly developing field of sustainability transformation(s).

The importance of the arts for transformations, while recognizing they are intermingled with that of the humanities, is outside the scope of this paper. The case of the arts has been solidly supported and illustrated by numerous recent academic publications (Alonso, 2015; Galafassi et al. 2018; Pearson et al. 2018; Muhr, 2020; Heras et al. 2021; Campbell et al. 2026; Strand et al. 2024; Chen and Mao, 2026); not least by the integration of arts in the work of the Anthropocene Working Group (AWG). The arts have known a surge in sustainability transformation research through the development of arts-based visioning and interventions associated with transformative and relational approaches to sustainability science and research more broadly (Asplund et al. 2023; Gianelli et al. 2024; Poliseli and Caniglia, 2024; Horvath et al. 2025).

## Sustainability transformation(s): a simple heuristic

For the purpose of this paper, we chose a definition of sustainability transformation proposed by the Intergovernmental Science-Policy Platform on Biodiversity and Ecosystem Services (IPBES) in its 2019 global assessment report: “fundamental, system-wide reorganization across technological, economic and social factors, including paradigms, goals and values,” expanded in 2024 with the IPBES Transformative Change Assessment to fundamental, system-wide shifts in views (“ways of thinking, knowing and seeing”), structures (“ways of organizing, regulating and governing”) and practices (“ways of doing, behaving and relating”) (IPBES, 2024a:5). While recognizing the limitations of these definitions, we motivate our choice as follows: (i) it explicitly includes more intangible aspects such as views, paradigms and values—traditionally the focus of the humanities—alongside more concrete structures and practices, and (ii) it may be closer to a scientific consensus than other definitions as it represents the result of intense inter- and trans-disciplinary deliberations among researchers and practitioners within IPBES processes. We also acknowledge the steps taken to integrate the humanities into the IPBES sustainability assessments, including the creation of the Social Sciences and Humanities Network (SSH Network https://onet.ipbes.net/node/43) community of practice at the end of 2020 (Stokland et al. 2022). For example, ~10% of the experts involved in the Values Assessment had an academic background in the humanities (Mäkinen-Rostedt et al. 2023).

**Fig. 1:**
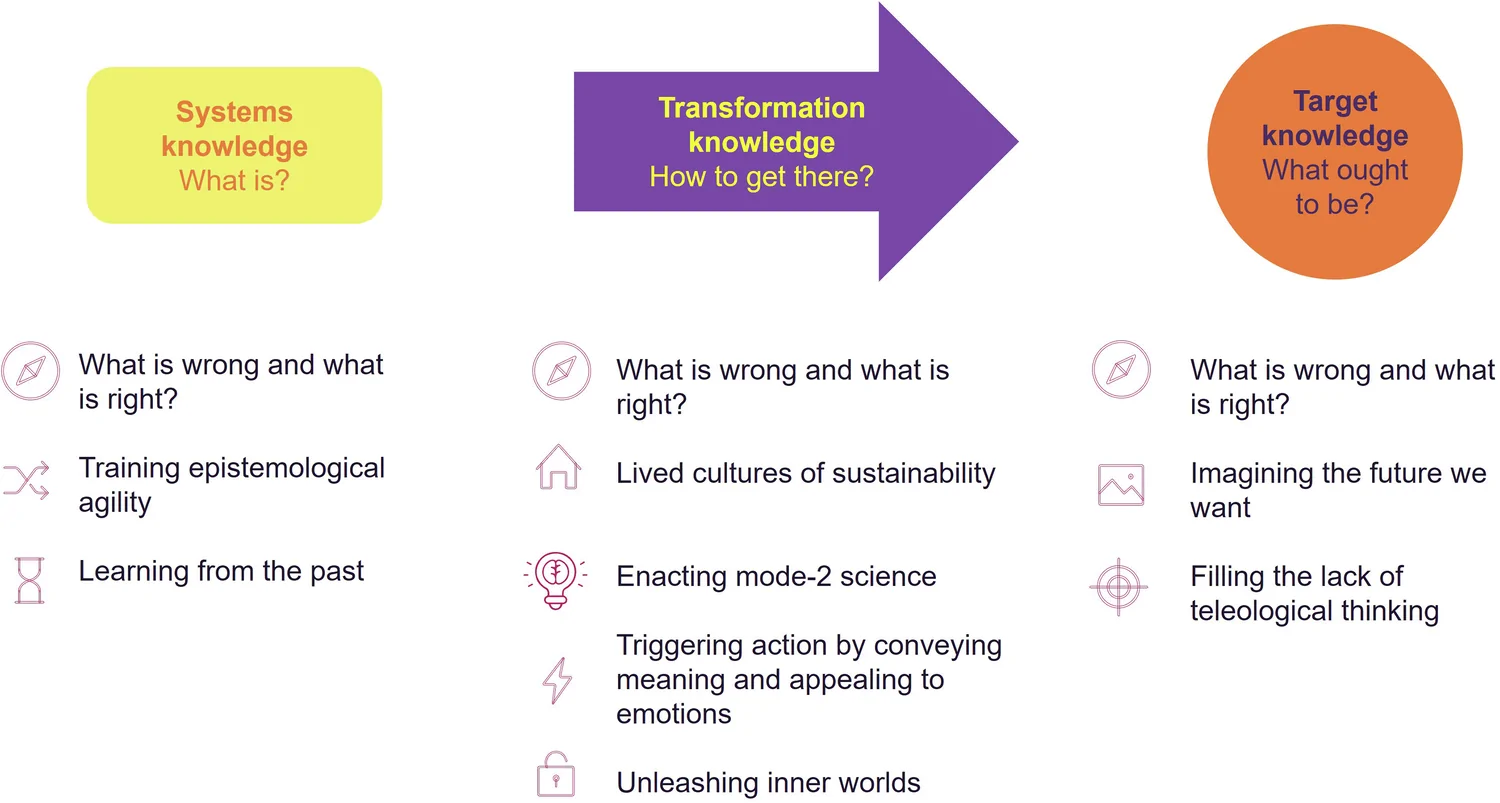
The contributions of the humanities to the three types of knowledge needed for sustainability transformation(s) as defined by sustainability science.

Three types of knowledge are needed, according to sustainability science, for sustainability transformation: (1) systems knowledge, an understanding of a system’s current dynamics and function; (2) target or normative knowledge, an understanding of a system’s desirable future vision(s); and (3) transformation knowledge, an understanding of the pathways and intervention strategies needed to reach that desirable vision (Fig. 1; Brandt et al. 2013; ProClim, 1997). Mainstream scientific contributions overwhelmingly observe, characterize, analyze, problematize and respectively predict the current problem constellation(s) and plausible future constellations at different scales (Scheffer et al. 2024). Most of the sustainability research to date has focused on past, present and future analytical descriptions of social-ecological systems of interest. Envisioning desirable futures at local and regional levels and especially reaching these futures have remained relatively underexplored in current sustainability research (Brandt et al. 2013; Baumann et al. 2023). So, comparatively, target and transformation knowledge have received considerably less attention, leading to persistent knowledge and approach gaps. We argue in nine points that the humanities, not limited to the environmental humanities, are well placed and uniquely suited to contemplate, investigate, and engage specifically with these gaps.

The contribution of the humanities to systems knowledge is widely recognized, owing to their descriptive and, in particular, their reflexive and critical competencies, i.e., the capacity to question norms, practices and one’s own assumptions and positionality, as well as to reflect on values, perceptions and actions (UNESCO, 2018) (see also points 1, 7, 9). In the same vein, when considered through the lens of key competencies for advancing sustainability transformations, the humanities not only support the general competency of critical thinking, but also make distinct contributions to transformation-oriented competencies such as values thinking (point 1), futures thinking (point 2), inter- (point 5), and intra-personal (point 6) competencies (Brundiers et al. 2021; Redman et al. 2021). Consequently, we primarily elaborate on how the humanities are shedding light precisely on target and transformation knowledge by addressing and challenging the dominant “how” of transformative change and by expanding the visions and “solution space” to a level of complexity commensurate with global sustainability challenges (Stirling, 2014; Shrivastava et al. 2020). In the following nine interrelated points, grouped according to the type of knowledge they chiefly contribute (see Fig. 1 for guidance), we illustrate how the humanities are well-equipped to support visions and pathways for sustainability transformation(s).

### 1. What is wrong and what is right? Asking foundational questions

The theoretical traditions rooted in the humanities are relevant for interrogating and confronting the foundational assumptions of modern societies, and for structuring the ethical and normative dilemmas that consumption-intensive societies face, including questions not only about what is, but also about what ought to be (Fischer et al. 2007). For example, examining the assumption of a universal undifferentiated ‘we’ from feminist, intersectional, post- and decolonial perspectives illuminates historical, social, and geopolitical asymmetries (Spivak, 1988; Mbembe, 2003; Karera, 2019; Ferdinand, 2022). At the core of the humanities lie the key, albeit uncomfortable, questions of humanity. Who are ‘we’ as humans, how do ‘we’ understand ourselves, which principles should guide ‘our’ actions, what is wrong and what is right, which are the things ‘we’ consider of the highest importance, what is truth, what is a good life, what is beautiful to ‘us’, what may responsibility and accountability look like in the Anthropocene (Chakrabarty, 2009, 2021; Horn and Bergthaller, 2020)? The humanities open a space of possibilities that moves beyond the present, the probable and the concrete to which political sciences often attend. Humanities interrogations extend to the future, the intent, the aspirational, and to the teleology (the goal-oriented reasoning about the future, see point 8), nudging ‘us’ to ask who do ‘we’ want to be, who do ‘we’ want to become today in a world that is fundamentally tested by a polycrisis, and what kind of relationship ‘we’ seek to cultivate with future generations —yet, who is included in this ‘we,’ and who decides on the terms of inclusion? (Sand, 2019; Oomen et al. 2022; Lawrence, 2024). These questions go beyond formal structures, institutional and procedural inclusion to make visible the invisible structures of thought and meaning in view of long-term and aspirational horizons. Once this reflection work is done, humans are equipped with a self-understanding that can fuel their efforts to overcome complex wicked problems, such as the climate change perfect moral storm (Gardiner, 2006).

One direct application of this reflexive understanding would be to gauge what a morally just sustainability transformation might require, and to articulate its ethical, historical, and philosophical terms, including revisiting the notions of accountability and responsibility in a distributed way. For example, world-making perspectives, building on constructivist accounts of multiple “world-versions” (Goodman, 1978) and further developed in posthumanist literature, offer a strong sense of responsibility to enact subjectivities and relationships in deliberate ways, emphasizing the tenet of everyone’s involvement in the process of world-making (Gram-Hanssen, 2026, see also points 6 and 8). This first step of deep reflection on normative questions and ethical aspects that underpin the human condition in the Anthropocene is rarely acknowledged when initiating a forward-looking transformation pathway or strategy (Palsson et al. 2013). Since the 1990s, the sustainability agenda has largely set aside engaging with normativity, yet sustainability is a normative values-driven concept. The consequence is a tendency to leap directly to action-oriented and techno-centric measures, and to compose transformation trajectories exclusively from socio-technological solutions, many of which operate under the label of technofixes addressing symptoms rather than root causes (Abson et al. 2017; Turnhout, 2024). Humanities perspectives make these underlying value commitments visible and interrogate epistemes that sustain extractive models of living and relating in which nature is reduced to a resource (Böhnert and Reszke, 2022). Indigenous governance approaches have reshaped decision-making principles through frameworks such as Free, Prior and Informed Consent and the Rights of Nature movement, as exemplified by the Whanganui River receiving legal personhood in 2017 (Charpleix, 2018; Bennett et al. 2024).

This point invites transformation scholars, whether entering from sustainability science or other fields, to begin with a deliberate interrogation of normative and ethical foundations that underpin sustainability challenges and visions before turning to the (co-)design of solution options, often through collaborative modes. These collaborative processes for sustainability transformations, i.e., knowledge co-creation and the involvement of diverse actors, carry their own ethical complexities. These may include commitment to sustainability-aligned values (Martin et al. 2024; Wittmayer et al. 2024), concerns to maintain accountability to both society and science, and to avoid reproducing unjust structures while necessarily working within existing ones (Horcea-Milcu et al. 2024). Decisions in knowledge co-production processes, such as whom to include and who decides on inclusion, can be supported by different ethical theories such as utilitarianism (stakeholders selected based on anticipated utility), deontological ethics (selection guided by moral goals, e.g., inclusivity), and virtue ethics (selection grounded in virtues such as humility) (Partelow et al. 2024), while an ethics of entanglement derived from the relational paradigm of new materialism can guide the relationships with participants (Haider and Rieser, 2025).

### 2. Imagining the future(s) we want: visions and narratives of transformation

There are branches of the humanities such as media and communication studies, linguistics, rhetoric and argumentation studies, which, based on prior research on argumentative strategies (persuasive patterns, fallacies, word choice), can forecast debate lines (e.g., Wang et al. 2017). However, by and large, the humanities are well placed not in the realm of the probable and predictable, but in the realm of imagining and reaching for desirable futures, identifying new visions and narratives for more just and sustainable futures for all. Literature and literary studies paint a picture of how the world might end if humanity crosses thresholds (or tipping points, in the language of global change research), and of the undesirable transformations that could occur if extractive models of living persist (dystopian futures, ecohorror, climate fiction). Conversely, the humanities support envisioning desirable futures and complement scenario simulations and predictions by addressing not only what is plausible but also what is desirable (Villasante et al. 2024), even that which cannot be observed empirically in the immediate reality (utopias of transformation). However, in practice, anticipatory research often focuses more on simulated plausible scenarios, anchored in past and present data and embedded in specific assumptions, than on desirable futures, which require normative, forward-looking perspectives (Nikoleris et al. 2020; Baumann et al. 2023; Juri et al. 2025). Consequently, predicting probable futures aligns with systems knowledge, envisioning desirable futures with target knowledge, and scrutinizing the performativity of imaginaries (sensu Muiderman, 2020) with transformation knowledge.

Between utopian visions and dystopian futures, the space of possibilities can be expanded to infinite alternative worlds and narratives (Luederitz et al. 2017; Fazey et al. 2018), also reflected in literary subgenres such as ecotopian solutions, fairy tale climates, solarpunk, cli-fi, Indigenous futurisms and ancestral dystopias (Whyte, 2018; Jenkinson, 2026). Enabling a different immaterial horizon to replace outdated narratives of human dominance of the planet and to acknowledge more-than-human and material agency often happens in the realm of the humanities (Ballard, 2021; J. Bennett, 2010; Tsing et al. 2017). The humanities help train the muscle of imagination, so necessary to contrast the status quo, but atrophied by dominant linear, mechanistic, technocratic thinking (Moore and Milkoreit, 2020, see also the conference Fantastic Climates). Palmer and Fam (2022) note that literature and arts permeate our inner worlds (see also point 6) and create moments of awe where reality in the Newtonian sense gets suspended, and different ways of being with the more-than-human world and of transdisciplinary collaboration appear possible. This is all the more important as many of the visioning exercises in transdisciplinary sustainability research and futures work are hampered by participants’ inertia or limited trained ability to use their imagination (Soria-Lara et al. 2021; IPBES, 2024b). In contrast, the humanities and especially the arts open up space for an emancipatory imagination of alternative futures. These futures entail the dismantling of racism, the redistribution of power, the fair sharing of burdens (e.g., health-related) and climate change impacts, and equal access to (environmental) benefits across racial, ethnic, Indigenous, migrant, and socially marginalized groups, in line with notions of racial and climate justice (Deivanayagam et al. 2023; Demos, 2023).

The humanities negotiate the basic conceptions of history, past, present and future, and their implications in navigating between what has been and what could be (Citton, 2010). As such, the present not only shapes the future, but concepts and visions of the future also influence the present through anticipation (Koselleck, 2003; Mische, 2009; Appadurai 2013). Studies of the great diversity of human and cultural organization in the past reveal the myth of apparently natural present social orders and offer space for alternative political and systemic imaginaries (Graeber, 2024). To shape a new future, the transition out of exploitative forms of living cannot happen without the power to reimagine visions of radical social change. The process of visioning can draw on the humanities to develop both new evolutionary discourses and metaphors to guide humanity’s journey across geological times (Shrivastava et al. 2020; Chabay et al. 2021; Holmén et al. 2026), as well as to adopt existing ones to convey hope and aspiration (Villasante et al. 2024). Many of these previously marginalized narratives of care and reciprocity, such as Indigenous ones, are explicitly relational (Staffa et al. 2022). Whether old or new, visions and narratives of transformation may represent starting points for better decisions or action incentives (Carpenter and Folke, 2006; Villasante et al. 2024).

### 3. Living cultures of sustainability

Cultures are collective meaning-making systems sitting at the interfaces between selves and others where they can strengthen or weaken continually unfolding connections among co-evolving individuals (Hall, 1997; Vilsmaier et al. 2017; Shrivastava et al. 2020; Wamsler et al. 2022). As integral parts of cultures, language and discourses act as dynamic and historical practices in which meanings and norms are continually reshaped through acts of speaking, thereby involving processes of cultural change (Coseriu, 2007). Cultures mediate a social meaning of sustainability and govern sense-making processes around it, while also manifesting in practice(s). Transferring sustainability from a concept to an embodied practice at the family and community level may be one transformative strategy for moving away from a global culture of non-sustainability to a whole plurality of Cultures of Sustainability (Kivitalo, 2017; Parodi and Tamm, 2018). The aggregated SDGs are hiding an incommensurable diversity of (operative) ways of being, doing and relating aligned with the ethos of sustainability. While cultures can pose a governance challenge to implementing the SDGs, they also push for their careful contextualization for different groups of people (Leal Filho et al. 2020). As relational concepts (West et al. 2024), cultures accompany and co-evolve with the spiritual, intellectual and aesthetic development of a certain way of living of a particular collective, understood more in an operative rather than intellectual or theoretical way (Heinrichs et al. 2016). Thus, the humanities hold that sustainability can gain traction and be operationalized at the level of human lived experience (including transformations) through culture and cultural embeddedness. Similarly, certain theories of transformative change view culture as permeating all three of Karen O’Brien’s (2018) spheres of transformation.

A culture of sustainability was initially defined as “a collectively supported, jointly agreed, and understandable sustainability which is institutionalized and internalized and is handed down through conventions, patterns, habits and even feelings” (Parodi et al. 2011:77). Cultures of Sustainability (CoS) were subsequently defined as “a narrative of embodiment and planetary consciousness” (Dieleman, 2017) and as lived practices, formal and informal processes which shape the shared norms, values and framework conditions towards sustainability of a community (Fritz et al. 2022). A culture of sustainability integrates ethics and aesthetics of living with moderation (Kagan, 2010; Vogt and Weber, 2019), and avoids disconnection: disconnection with others, with the more-than-human, with self and with something higher, recognized among the root causes of sustainability crises (Wamsler et al. 2022). It therefore redefines human well-being away from consumerism and into the quality of relationships between human and other-than-human, between generations, between self and others and even into the quality of the relationship with the self (Wamsler et al. 2022). Chapter 5 of the IPBES Transformative Change Assessment gives an account of Indigenous and relational philosophies characterizing human-nature relationships across diverse cultures, amenable to cultures of sustainability (Gosnell et al. 2024). For example, Buen Vivir is integrated into the constitutional frameworks of Ecuador (Delgado Ramos, 2014). Meanwhile, emerging CoS include sustainability as an acknowledged part of human well-being and of a good quality of life (Glynn et al. 2025).

Cultures of Sustainability, complementary to rules, regulations and ‘policies of sustainability’, allow for a certain autonomy of sense-making and for embracing cultural identities (Glynn et al. 2025). Hence, CoS fosters a collective sense of agency, which paves the way for people to find their own sustainability in harmony with their normative, cognitive, and emotional experiences (Werlen et al. 2026). As cultures continually propagate and replicate themselves, the humanities ask how sustainability can be embedded endemically within cultural practices, contextualized and integrated into everyday life, rather than imposed solely through top–down national targets. Part of the answer is that CoS can be catalyzed by new, strong stories, images, sensations and languages of desirable futures (see point 2 above), thereby becoming an impulse for collective action. In keeping with the recommendation of the German Rectors’ Conference (HRK, 2018), the voluntary association of 271 German universities representing them in political and public affairs, advocating for the establishment of a culture of sustainability at universities, the University of Kassel created a Cultures of Sustainability Department as part of the newly founded Kassel Institute for Sustainability. In the same vein, the Institute for Technology Assessment and Systems Analysis (ITAS) established the Karlsruhe Transformation Center for Sustainability and Cultural Change (KAT). Moreover, the German Federal Ministry of Education and Research funded the project “Universities in society—Real-world experiments of transformative learning and research for a culture of sustainability” (KuNaH, 2022-2025), while the German Federal Environmental Foundation regards the cultural sector as the fourth pillar of sustainability (Nunes et al. 2017; Bonde and Fuhrmann, 2021).

### 4. Close to mode-2 science

New and collaborative modes of knowledge production are needed if science is to serve as a lever for achieving visions of just and sustainable futures. One of the most useful ways to envisage a more socially engaged role for science and to rethink the science-society and knowledge-action relationships is to seek to distinguish between mode-1 and mode-2 of knowledge production. Mode-1 science uses established scientific methods to generate disciplinary knowledge. Mode-2 describes a reflexive, dialogical science-society relationship, in which knowledge is produced in ‘the context of application’ and is often co-created transdisciplinarily with a variety of societal actors. Mode-2 science is characterized by a great diversity of the sites at which knowledge is produced (Nowotny et al. 2003) and does not rigorously adhere to the ideal of generating universal knowledge (Langemeyer and Zimpelmann, 2023). The quality of the produced knowledge is also evaluated using social accountability criteria such as relevance and usability. Consequently, mode-2 science is relevant and accepted in sustainability transformations research (at least in its transformative branch, Thoren and Breian, 2016), and also one of the key references of the transdisciplinarity discourse (Jahn et al. 2012; Rigolot, 2020).

Notably, within the disciplinary landscape commonly foregrounded in sustainability transformation research, the humanities appear more closely aligned with mode-2. In their book on the new production of knowledge, Gibbons et al. (1994) dedicate a whole chapter to “the case of the humanities”. The humanities, as a diverse field and mode-2 science, as a historical development of science cultures and contexts (Langemeyer and Zimpelmann, 2023), appear to exhibit certain shared characteristics highlighted by Gibbons et al. (1994). For example, “they do not only exhibit reflexivity in a more continuous and radical manner than the natural sciences. Their reflexivity is expected to carry meaning for the entire human experience” (Gibbons et al., 1994:92). Unlike the natural sciences, the humanities cannot rely on cumulative, species-level explanations that merge human history with biological evolution. Such accounts overlook the historical and social differences that shape human experience. “While the social sciences attempt to keep some kind of distancing from what they observe and interpret, in large parts of the humanities and in cultural production no such aloofness is observed to delineate or express meaning” (Gibbons et al. 1994:92).

In line with their resistance to narrow definitions, transdisciplinarity is present across areas of the humanities in which disciplinary boundaries are loose and organized at the level of professional microcultures sharing intellectual affinities, but also contradictions in interpretations (Gibbons et al. 1994). Quality in the humanities is not subject to peer control only, but to external diverse validation, such as interpretive coherence or cultural sensitivity (Kagan, 2009). These quality criteria suggest adaptability towards the mission-driven nature of mode-2 science. As per mode-2, methods grounded in the humanities and based on lived experience, such as first-person methods, phenomenology analysis (Frank et al. 2024; Woroniecki et al. 2024), are suited to experiment with innovative and progressive approaches, as demanded by the unprecedented sustainability challenges. The humanities can entertain the organization of the knowledge co-creation process as an intervention that facilitates intentional change toward desirable future(s) in a contextually defined system (Horcea-Milcu et al. 2024; Marciniak et al. 2024, see also point 5). Mutual and continuous learning between academia, industry and society is seen as an important precondition and outcome of these collaborative processes or interventions, because both mode-2 science and the humanities consider that knowledge is fluid, and education is life-long beyond formal schooling (Chabay et al. 2021).

### 5. Inspiring transformations and triggering action by conveying meaning and appealing to emotions

Facts are necessary for a transformation, but not sufficient (Böhnert and Reszke, 2022). Evidence-based knowledge about the sustainability crises has been available for decades, yet it has not translated into the scale and speed of political and societal transformations required to face the challenges of the Anthropocene (Stoddard et al. 2021). Yet, humankind is quicker to adapt and evolve through cultural processes rather than biological processes. The tools of the humanities, such as storytelling, message framing, metaphors, linguistic expressivity and aesthetic experiences, have a role in delivering a message of urgency and hope (Moriggi et al. 2020; Bentz et al. 2021; Poliseli and Caniglia, 2024). Performative methods can actively engage people in transformation processes (Heras and Tàbara, 2014). Through the power of words, the humanities can convey to people that they indeed matter (O’Brien and Hochachka, 2024, see also point 6). Technological solution options that lack cultural embeddedness or complementarity with other approaches remain mere band-aids (see point 3). They must be implemented and communicated in ways that people can understand and integrate into their lived realities (e.g., NIMBY responses to wind turbines or resistance to heat pumps). By co-producing cultures, the humanities help shape lifestyles and values, thereby enabling behavioral change and strengthening the societal traction needed to foster Cultures of Sustainability.

Regarding how to communicate about sustainability transformation(s), the humanities' contribution is twofold. On the one hand, they provide a variety of media, such as spoken, written or multimodal discourse, to express meaning or inspire (collective) action. Narrative-based climate engagement demonstrates how literary and cultural analyses of stories and imaginaries are used in community climate processes to shift perceptions of responsibility and possibility (Oomen et al. 2022; Yusoff and Gabrys, 2011, see also point 2). Culturally sensitive framing increases the likelihood that messages will build trust and acceptance, especially because transformation models must resonate with entire societies, not only with experts (Schwarz-Friesel, 2013). Matters of concern, such as lack of trust, tend to be put into words and communicated about, especially when they are missing (Böhnert and Reszke, 2022). Moreover, the humanities offer much-needed vocabulary for knowledge mediation through culture and aesthetic experiences. Artists and authors translate propositional knowledge (a dominant form of knowledge involving factual “that is” knowledge, often seen in opposition with “know-how” knowledge) on anthropogenic climate change a through distinct visual and narrative strategies that underscore the necessity to reconsider non-propositional knowledge (Remmers, 2015; Zingelmann, 2024) in transformative and transformation sustainability research (Horcea-Milcu et al. 2024).

On the other hand, deconstruction strategies grounded in the humanities lay bare bias, miscommunication and manipulation. Political, commercial and media discourses instrumentalize or even weaponize certain concepts and worldviews, manipulating inner worlds through processes which may feed the polycrisis (Bristow et al. 2024). The humanities can identify and pinpoint unreasonable doubt or historical legacies and toxic narratives that perpetuate injustices (Wals, 2015; Franklin, 2020). For example, linguistic analysis can be an antidote to conspiracy theories (Stumpf and Römer, 2024). Similarly, the humanities provide references and resources for creating reflexive identities, in contrast to the collectively imposed identities built through advertising and media manipulation (Gibbons et al. 1994). For example, the Greek tragedy genre still offers the possibility today to reflect on ethical dilemmas, socio-psychological implications, and political responsibilities posed by anthropogenic climate change. Humanities may offer a moral orientation and normative landmarks (see point 1) which are transmitted in ways easier to accept than authoritarian, control-based and top-down ones, as they appeal to inner worlds (see point 6 below).

### 6. Acknowledging and empowering the self: unleashing the inner dimensions of sustainability transformation

Transformation research has predominantly focused on outer worlds, external dimensions of reality such as economic systems, biophysical parameters, technofixes or behavioral metrics, often at a planetary scale (Shrivastava et al. 2020). Inner dimensions of reality at the individual and collective levels, such as affects, deeply held values, and paradigms, have most often been left outside the discourse on how to reach a sustainable future. In addition, approaches based on global solutions risk remaining mechanistic, technocratic, and control-based, while ignoring how transformations are made sense of and experienced by people at a personal level (Woroniecki et al. 2024). Recently, researchers have made progress in addressing these blind spots, acknowledging notions such as the personal sphere of transformation, personal sustainability and inner transformation. “The personal sphere of transformation represents the subjective beliefs, values, worldviews and paradigms that influence how people perceive, define or constitute systems and structures, as well as their behaviors and practices” (O’Brien, 2018:156). “Inner transformation is about the powerful unleashing of human potential to care, commit to, and effect change for a better, more sustainable life across individual, collective and system scales” (Wamsler et al. 2021; Ives et al. 2023). Inner transformations for sustainability rose as a sizable research topic of the journal Sustainability Science (Sahle et al. 2025) and were recognized as one of the six broad approaches to transformative change by the IPBES (Bennett et al. 2024). In line with “people as solutions to sustainability” (O'Brien and Hochachka, 2024), a report convened by members of the Club of Rome introduces four elements of inner worlds: meaning-making systems (worldviews, paradigms, mindsets), values, identities, and spirituality forms (Bristow et al. 2024). Non-profit organizations joined efforts and developed a framework for inner development skills and capacities (being, thinking, relating, collaborating and acting) called the Inner Development Goals. All these initiatives converge on the idea that empowered inner worlds can counteract status quo levers from the outer world.

The humanities are well positioned to investigate and activate these more and more identifiable overlooked dimensions: “the personal side, the micro level, the conscious and acting subject and inner human world” (Parodi and Tamm, 2018:12). They are more apt to deal with the dualities, contradictions and paradoxes inherent to human inner worlds, and not ’solve’ them through a separation and fragmentation either|or approach (see point 4). Research-wise, inner worlds can be studied using first-person methods of inquiry and phenomenological analyses into subjective experiences (Frank et al. 2024; Woroniecki et al. 2024). While the social sciences explain human behavior through values as a proxy for inner worlds (systems knowledge), the humanities can make the leap from understanding to intervention, mobilizing values and inner worlds (a transformation knowledge question, see also Horcea-Milcu, 2022; Horcea-Milcu et al. 2024). From an interventional stance, inner dimensions of sustainability can be activated, for example, through experiences, consciousness practices or transformative learning tools. Aesthetic, literary and spiritual experiences grounded in the humanities can enrich inner worlds by awakening hope and faith (Ives et al. 2023).

Consciousness practices include contemplative, psychological, facilitation and coaching, and education and leadership approaches (Wamsler et al. 2022). In her book “Radical Transformational Leadership”, Monica Sharma introduces a new field of “radical systems and cultural transformation”, as well as leadership techniques for integrating the power of inner worlds in outer systems change, such as the Conscious Full Spectrum Responses, which she considers a fractal of large-scale transformations (Sharma, 2017). Many of the mind-body contemplative practices and interventions, such as mindfulness, journaling, storytelling, prayer, visualization and other forms of training awareness and self-reflection (Wamsler et al. 2022) are grounded, among others, in cultural and religious traditions which fall in the competency domains of the humanities. Such interventions have, for example, been applied in contexts such as urban planning. In Auroville, a humanities-inflected approach, specifically the philosophy of Sri Aurobindo, shapes an alternative model of urban living that emphasizes community, inner transformation, and shared values (Woiwode and Bhati, 2019). Another application context is international climate negotiations, where experimental and safe micro-spaces were created (Wamsler et al. 2020; Mar et al. 2021). These bounded yet purposeful manifestations of desirable futures enable, through shared self-reflection, a different type of individual agency (Wamsler et al. 2020). Indeed, the humanities complement the collective-level political science perspective on agency by foregrounding interiorized consciousness-based dimensions. This points to a fractal logic of transformation, whereby values, practices, and forms of agency cultivated at the personal level can replicate and scale across contexts and levels, linking inner change to collective and systemic shifts (Ramcilovic-Suominen, 2024).

### 7. Training epistemological agility and seeing the epistemological landscape

A certain literacy in notions of theory of science and philosophy of science is necessary if science is to support academia’s “responsibility to inform and accompany transformations supporting just and fair futures for people and nature” (Marciniak et al. 2024:2; Nagatsu et al. 2020). Within collaborative interdisciplinary teams aiming to contribute to societal transformation, scientists need to clarify their own epistemological stance and assumptions regarding the role(s) of science (Ligtermoet et al. 2025), potentially drawing on concepts such as “thought collectives” (a group sharing non-epistemic and epistemic claims about truth, Fleck, 1979; Soininen et al. 2022), “situated knowledges” (all knowledge being shaped by the knower’s position, Haraway, 1988), “epistemic cultures” (arrangements used to validate knowledge; Knorr Cetina, 1999), “Matters of Concern” (issues entangled with values, politics, and stakes; Latour, 2004). Many of the factors hampering scientific collaborations and co-production, which are so vital for tackling sustainability challenges, can be explained by researchers entering from unaware epistemological starting points and unsurfaced backgrounds.

Humanities disciplines such as philosophy of science and science studies can map the landscape of existing epistemological starting points and, at the same time, facilitate a more nuanced understanding of how different knowledge systems may work in collaborative interventional research (Poliseli and Caniglia, 2024). For example, philosophical orientations range from viewing knowledge acquisition as value-free and generalizable to viewing it as value-laden and contextually unique (Moon and Blackman, 2014; SES-Link, 2017). Similarly, there are substantialist strands of sustainability science but also relational ones, which seek to transcend the nature-culture dualism by recognizing perpetual states of ‘becoming’ and continually unfolding processes in the world, in dialog with the (environmental) humanities (West et al. 2020; Blanco et al. 2025; Hertz et al. 2025). Other distinctions grounded in the history and philosophy of science and relevant for clarifying how science can enable social transformations are between mode-1 and mode-2 science (Gibbons et al. 1994, see also point 4), or between the transformative branch of sustainability science and the descriptive analytical branch (Wiek and Lang, 2016). A lack of awareness of the range of possible entry points, available epistemologies (assumptions about how knowledge is created) and ontologies (assumptions about the nature of reality) can hinder not only research collaborations, but also interactions and relationships between Indigenous and scientific knowledge holders (Rathwell and Armitage, 2015). Illustratively, Blaser (2014) argues that Indigenous peoples don’t just have different beliefs about the world, but enact entirely different worlds, contributing to a pluriverse (Escobar, 2018) of ontological plurality, coming into being through worldings such as the narrations of their own existence. These alternative worlds can also be actively cultivated through design for transitions practices (Escobar, 2018). The implications of such diverse epistemological and ontological dimensions of transformation (research) can be surfaced through targeted training and practice (Moon et al. 2021; Hakkarainen, 2022), before progressing to more integrative processes. In this regard, humanities-rooted assemblage theory (initially developed by Deleuze and Guattari, 1987) offers a postmodern alternative to classical approaches of transdisciplinary knowledge integration (Lang et al. 2012; Hertz and Bousquet, 2025). An assemblage, as a collection of radically heterogeneous things emerging into “doings”, resists the reduction of complexity (Hertz and Bousquet, 2025).

Within the multidisciplinary sustainability research communities about and for transformation, epistemological agility, understood as the ability to use different epistemological lenses (Haider et al. 2018), is needed in order to map and especially navigate the space of onto-epistemological positionalities potentially suitable to engage with a certain wicked problematique (Ligtermoet et al. 2025; McGowan et al. 2014; SES-Link, 2017). For example, epistemological agility is required to recognize the circumstances or cases in which research calls for a mode‑1 or mode‑2 standpoint, while remaining open to the interconnections between the two (Langemeyer and Zimpelmann, 2023). Engaging with values as leverage points lends itself to mode-2, while studying values as predictors of human behavior calls for mode-1 (Horcea-Milcu, 2022). Indeed, sustainability scientists are often expected to shift fluidly between disciplines and roles (West et al. 2020). Moreover, in the context of multidisciplinary and international collaborative knowledge production, it has been demonstrated that sustainability researchers can shift their epistemological worldviews (post-positivism, pragmatism, transformativism, constructivism in Creswell, 2014) over time (Hakkarainen et al. 2020; Mäkinen-Rostedt et al. 2023). Conversely, disciplinary evaluation panels should not perceive interdisciplinary humanists as a threat to disciplinary identity, as has been empirically observed (Salmela et al. 2025). Building on the above humanities contribution (and on point 1), transformation scholars can cultivate a practice that is more self-aware, more attuned to the diversity of doing transformation research, and more capable of maintaining epistemological agility.

### 8. Breaking the chains of causality with teleology

Unsustainability is also rooted in a lack of teleological thinking, characterized by its focus on direction, aim and purpose (Davelaar, 2021). A cause-and-effect logic has dominated knowledge production and decision-making, leaving almost no room for deliberate, intentional choices that shape outcomes (Fischer and Riechers, 2019; Davelaar, 2021). Yet, the goals and paradigms of a system, recently grouped under the system characteristic of intent, are considered deep and effective intervention points, whereas shallow leverage points belong to the realm of causality (Abson et al. 2017; Woiwode et al. 2021). It is worth noting that the humanities emphasize a distinction between teleological thinking and teleological historiography, as the former does not assume that history will unfold in predetermined periods and phases of birth, growth, maturity and decline. Rather, it acknowledges the multiplicity of possible paths, amongst which the most suitable to present knowledge is pursued. Teleological thinking complements the linear “knowing” under systems knowledge with the willing, the committing, the intending, the consciously choosing, necessary to build transformations and especially target knowledge.

From a teleological perspective, envisioning a desirable future (see point 2) makes the vision a telos. It speaks about articulating a goal, forming an intent, committing to a purpose and making a deliberate choice. Most approaches to sustainability transformations, including practices that seek to activate inner dimensions of transformations (see point 6), can occupy a place along a spectrum between causal and teleological orientations (Woiwode et al. 2021), i.e. between a stance in which change is explained primarily in terms of causal processes and one in which change is intentionally brought about. It is the latter that teleological thinking emphasizes. Teleology opens doors to a sense of deliberate purpose and intentionality that individuals, collectives, communities and maybe societies can adopt, ideally embody. At the same time, any given telos can be subject to renegotiation and change in light of new findings. Coupling intentionality and purposefulness with ethical and normative foundations (see also point 1) gives rise to a needed immaterial moral horizon (Sala and Torchio,  2019). A mindset of intentionality towards sustainability cultivates consciousness and opens the pathway; whereas path dependencies, linear causal reasoning and a mindset of optimization tend to legitimize short-term solutions that prioritize safeguarding existing power structures, extractive relations, and hierarchies inherited from colonialism (Cooper and Gibson, 2022).

### 9. Learning from the past to understand the present

The Anthropocene implies a full catalog of questions for the humanities, independently of its formal recognition, or ultimate rejection,as a geological epoch by stratigraphy. Central queries address the identification of the *Anthropos* in light of global and local social inequalities, histories of colonialism, and various forms of exclusion, not limited to racism and sexism (see also point 1): Who is the human in the historically situated world-making in the Anthropocene? Which notions of responsibility have been developed? Where does the nature-culture divide originate, and whate are its epistemic and aesthetic implications? At which spatial and temporal scales are humans able to witness their (historical) impact on Earth? Which past politics and esthetics have played a role in shaping our current environmental relations? How does our relationship with history shape our imaginaries for the future? Which existent environments and cultural objects are we able to and ought to conserve for the future, which ones ought to be discarded? While these questions may not be conclusively answered, they serve as descriptors of the potential of historic perspectives to contribute to systemic change and environmental conservation (Chakrabarty, 2009, 2021; Hall, 2019; Horn and Bergthaller, 2020; Scherer, 2022; Honeybun-Arnolda et al. 2024). Learning from the past explains how the present has been shaped as a result of historic events and gradual processes. This serves as an aid for orienting oneself within temporal developments and for identifying a telos (see also point 8).

Historic understanding must not be understood as normative, but rather as the processual reconfiguration of our narratives of the past and the constant assessment of present states. From decolonial and Indigenous perspectives, such historic understanding must also resist implicitly linear conceptions of time, instead foregrounding plural and historically grounded temporalities. In these, past, present, and future are co-constitutive, and reconfigured through situated practices of world-making guided by ancestral knowledge and responsibilities to future generations (Whyte, 2017; Scobie et al. 2025). As Honeybun-Arnolda et al. (2024) argue, world‑making in the Anthropocene arises through “ecologies of co‑production”, i.e., diverse, interrelated practices shaped by histories of exclusion, power, and institutional configurations. Their emphasis on history, relationality and multiplicity underscores that negotiating the telos with the immediate past means assessing whether the determined telos (point 8) and recent history align. This is crucial feedback for teleological thinking beyond mere quantitative evaluation. Even more so, it affirms the humanities' potential to embrace ambivalence. The historical analysis of cultural production during transformation, much like the ecologies of co‑production concept, invites insight into how deep change seeps into cultural identities, or whether cultural resistance, including poetic and aesthetic expressions, dictates the design of transformative strategies.

History, philosophy, cultural studies, postcolonial studies, and critical theory examine how epistemes, ideologies, and forms of decision-making and policy-making, some of which trace back to the European Enlightenment, have led to present sustainability-related wicked problems or reinforced patterns of unsustainable behavior. Narratives of human-nature relationships (e.g., human mastery of nature) and their historical contingency have shaped power structures that persist today. By contrast, relational understandings of nature as mother, kin, partner, giving parent, or wise elder, found in many non-Western Indigenous conceptions (e.g., Buen Vivir, the Sub-Saharan Ubuntu, or the Andean Pachamama) (Vasquez-Fernandez and Ahenakew pii tai poo taa, 2020; Gosnell et al. 2024) may offer resources for addressing contemporary challenges. Indeed, such relational ontologies are increasingly considered potential endpoints (i.e., visions, see also point 2) of transformation pathways (Villasante et al. 2024). Historical and literary analyses uncover the multiplicity of social arrangements that have been tried across different times and crisis contexts (Graeber and Wengrow, 2021), offering valuable perspectives on how societies might respond to contemporary and future challenges (Holm et al. 2015). World regions with colonial pasts view the European Enlightenment legacy with extreme ambivalence and articulate justified doubts about the promise of enlightenment (Amselle and Diagne, 2018). A retrospective analysis of how human agency, accountability and responsibility were understood, practiced and distributed in the past may outline more suitable ways for the future (Wyborn et al. 2021), potentially implying a rethinking of the Enlightenment. This Enlightenment reloaded, or Second Enlightenment (Göpel, 2016), may disrupt dualistic thinking, mark a reintegration of inner worlds, and redefine a good quality of life.

#### Limitations and conclusion

The humanities address one of the most persistent blind spots in transformations research: the generation of normative and transformative knowledge, as well as the densification of systems knowledge. Historical and literary analyses, as well as explorations of normative and ethical dimensions, contextualize empirics, complement the explanatory, analytical and technical strengths of natural and social sciences (systems knowledge). By engaging with utopias, ambivalence, and teleological thinking, the humanities enable us to envision desirable futures and move them closer to world-making, while learning from past successes, failures, and blind spots (target knowledge). Intentional interrogation of foundational assumptions, deliberative philosophical reflections, and aesthetic practices can inspire interventional tools that help emancipate relationships of power and exclusion, and shape transformative pathways (transformation knowledge, see also Sorman et al. 2026).

Transformations are both external and internal, structural and relational, systemic and personal (Bennett et al. 2024). All available options for knowledge production are needed in order for science to take an active role in processes of societal change. Mobilizing the humanities' entry points and scientific worldviews for how transformative change happens is not the only key, but forms part of the responses of science and society to sustainability crises (Scheffer et al. 2024). To mitigate the risk of fragmented responses, science needs to allow diverse modes of knowledge production to coexist. While being aware that there is no clear-cut distinction between the contributions of humanities scholarship to transformations and those of other disciplines, the plurality of approaches available to tackle the polycrisis needs to include the distinctive ways of the humanities: reflexive, culturally attuned, emotionally resonant, and ethically defensible (Palsson et al. 2013; Holm et al. 2015; Gabriel et al. 2022). On the one hand, sustainability scientists and practitioners can become aware of humanities-based stances to expand the solution-options space, challenge path dependencies, and embed transformations in lived cultures. On the other hand, the humanities face the challenge of not automatically dismissing the more reductionist cultures of thought, but rather stepping alongside other disciplines to guide humanity toward more just, sustainable, and culturally grounded futures.

## Data Availability

All data supporting this paper, i.e., interview notes from eight semi-structured interviews, are available from the corresponding author upon reasonable request.
